# Supplementary material for: What does it take to learn the rules of RNA base pairing? A lot less than you may think
Source: bioRxiv. 2025 Aug 2:2025.07.31.668042. Preprint. [Version 1] doi: 10.1101/2025.07.31.668042 (PMC12324431; doi:10.1101/2025.07.31.668042)

WARNING: R2R is not intended to evaluate evidence for covariation or RNA structure where this is in question. It is not appropriate to use R2R´s covariation markings to declare that there is evidence of structural conservation within an alignment. R2R is a drawing program. As the original paper, Weinberg and Breaker, 2011 wrote:
"This automated R2R annotation [of covariation] does not reflect the extent or confidence of covariation. While such information can be useful, we believe that thorough evaluation of covariation evidence ultimately requires analysis of the full sequence alignment. For example, misleading covariation can result from an incorrect alignment of sequences, or from alignments of sequences that do not function as structured RNAs. Unfortunately, there is no accepted method to assign confidence that entirely eliminates the need to analyze the full alignment."
To disable this warning, run r2r with --disable-usage-warning

manA-pknot1.cons
subfam_weight=0.891987

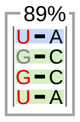

manA-pknot2.cons
subfam_weight=1

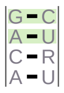

manA-var1.cons
subfam_weight=0.028518

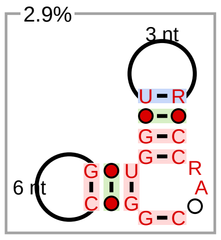

manA-var2.cons
subfam_weight=0.891987

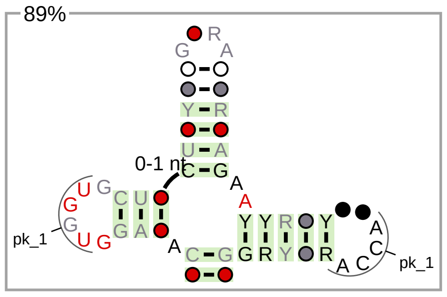

manA-var3.cons
subfam_weight=0.0794949

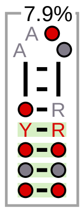

manA.cons

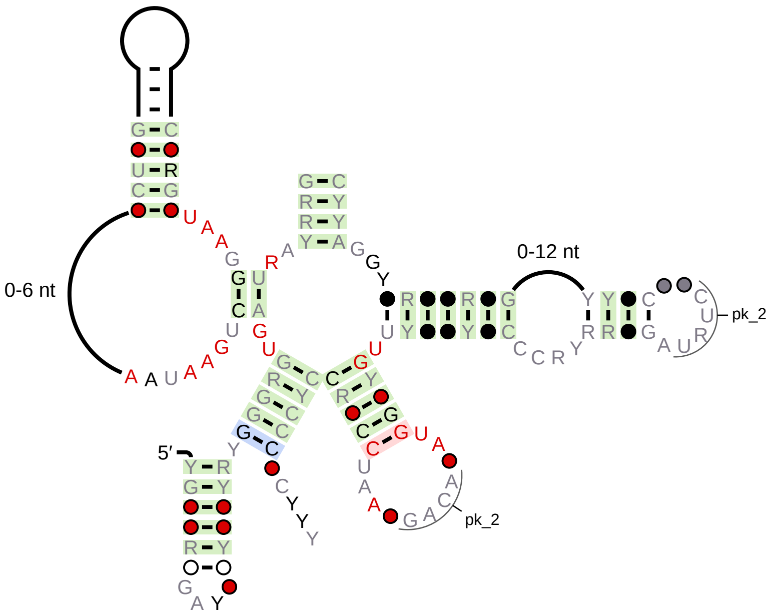

manA.cons NZ_AAOJ01000001.1/468755-469351

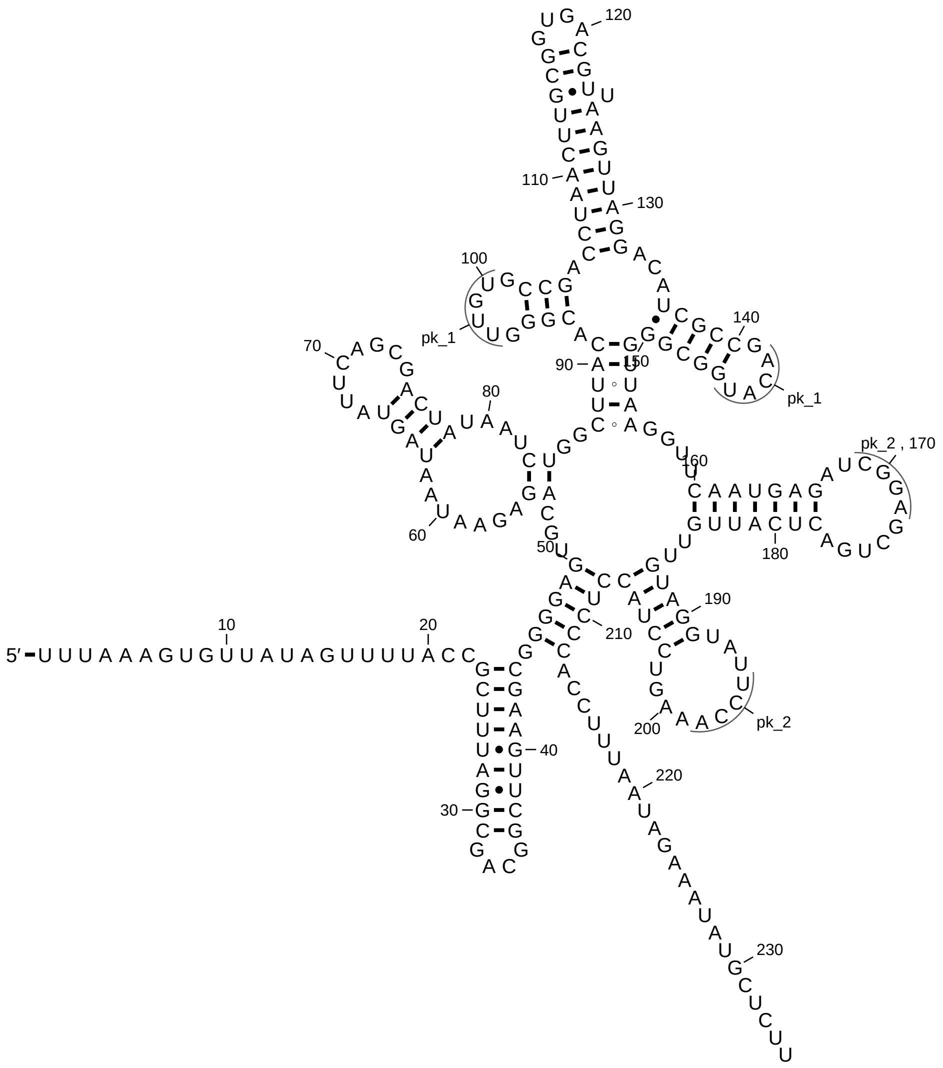

manA.cons skeleton varhairpin=1 var-right-stem=1

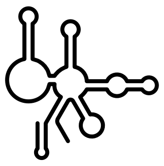

manA.cons skeleton-with-bp varhairpin=1 var-right-stem=1

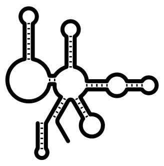

manA.cons varhairpin=1 var-right-stem=1

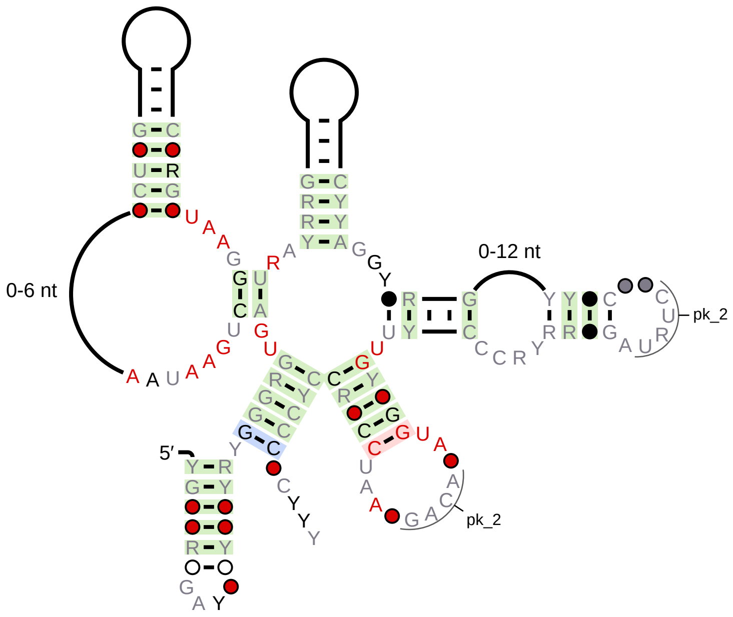

Supplement: Supplement 1 [file media-1.gz › supplemental_material/src/rscape_v2.6.0.tar.gz!/rscape_v2.6.0/lib/R2R/R2R-current/build-package/R2R-1.0.6.cand7-1/demo/output-pdf/manA.pdf]
